# Contemporary knee arthroplasty: one fits all or time for diversity?

**DOI:** 10.1007/s00402-021-04042-4

**Published:** 2021-07-16

**Authors:** Johannes Beckmann, Malin Kristin Meier, Christian Benignus, Andreas Hecker, Emmanuel Thienpont

**Affiliations:** 1 Department of Endoprosthetics, Sportklinik Stuttgart, Stuttgart, Germany; 2 Department of Orthopedic Surgery and Traumatology, Inselspital, Bern University Hospital, University of Bern, Bern, Switzerland; 3 Cliniques Universitaires Saint-Luc, Bruxelles, Belgium

**Keywords:** Partial knee arthroplasty, Unicondylar knee arthroplasty, Patellofemoral knee arthroplasty, Bicondylar knee arthroplasty, Customized knee arthroplasty

## Abstract

**Introduction:**

Total knee arthroplasty (TKA) has historically been the preferred solution for any type of knee osteoarthritis, independently of the number of compartments involved. In these days of patient-specific medicine, mono-compartmental disease could also be approached with a more individualized treatment, such as partial knee arthroplasty (PKA). Off-the-shelf (OTS) implants are often the compromise of averages and means of a limited series of anatomical parameters retrieved from patients and the pressure of cost control by limited inventory. Personalized medicine requires respect and interest for the individual shape and alignment of each patient.

**Materials and methods:**

A Pubmed and Google Scholar search were performed with the following terms: “patient-specific knee” and “arthroplasty” and “custom implant” and “total knee replacement” and “partial knee replacement” and “patellofemoral knee replacement” and “bicompartmental knee replacement”. The full text of 90 articles was used to write this narrative review.

**Results:**

Unicondylar, patellofemoral and bicompartmental knee arthroplasty are successful treatment options, which can be considered over TKA for their bone and ligament sparing character and the superior functional outcome that can be obtained with resurfacing procedures. For TKA, where compromises dominate our choices, especially in patients with individual variations of their personal anatomy outside of the standard, a customized implant could be a preferable solution.

**Conclusion:**

TKA might not be the only solution for every patient with knee osteoarthritis, if personalized medicine wants to be offered. Patient-specific mono-compartmental resurfacing solutions, such as partial knee arthroplasty, can be part of the treatment options proposed by the expert surgeon. Customized implants and personalized alignment options have the potential to further improve clinical outcome by identifying the individual morphotype and respecting the diversity of the surgical population.

## Introduction

Total knee arthroplasty (TKA) might no longer be the only suitable therapeutic option for each patient presenting with some type of knee osteoarthritis (OA). Usually, knee OA starts in one of the three knee compartments. Its aetiology is multifactorial and includes increased forces through the knee joint, either due to obesity [1] or because of alignment deviations [2–4]. Also anatomy modifying events such as previous trauma [5, 6] and total meniscectomy [7, 8] have been described as risk factors for the development of knee OA [1]. Depending on the degenerative status of the non-affected compartments and the philosophy of the treating surgeon, total knee arthroplasty (TKA) can be considered as an overtreatment. The times of a “one-fits-all approach”, where all patients despite of age, gender, ethnicity and activity level, receive the same treatment are over. Besides the obvious differences in size between human beings, their demands and expectations might be different. The promising idea behind personalized medicine is to tailor the therapy for each individual person. While personalized medicine has already gained a foothold in other fields such as oncology, it is time to implement personalized medicine in orthopaedics as well.

Mono-compartimental OA can be an excellent example for this more individualized approach by treating the patient with a unicompartimental knee arthroplasty (UKA). The ligament and bone sparing character of this resurfacing procedure makes UKA an attractive solution, especially for young patients. In addition, better performance and less mortality after UKA have been reported [9–12]. However, the usage of UKA is still behind that of TKA. PKA reports lower survival in the different national registries and a three times higher revision rate. This more delicate surgery needs a more detailed understanding of indications and a more refined surgical technique, both asking for experience. However, the most frequent number of UKA done according to the UK register by each surgeon each year is 1, followed by 2 [13]. The need for experience in every profession is evident and has been well described over the past years as a need for a higher percentage of UKAs within the surgeon’s case mix [12, 14, 15]. Therefore, for the first time ever, a registry could show non-inferiority in revisions of UKA in hospitals with high usage [16].

But assistive technologies could be a proxy for surgical experience, which asks for time and patient volume. Besides choosing the optimal surgical approach for each individual patient, many technical factors can lead to lower failure rates, such as accuracy of cuts and component positioning, fixation techniques and advanced gap balancing. Because of the high variability in patients’ individual anatomy, the use of an off-the-shelf implant with standard dimensions results in the adaption of the individual patient’s anatomy to the implant, requiring excessive bone resections, the creation of symmetric gaps and the need to adapt the soft tissues to the structural modifications. Patients with an outlier anatomy will certainly benefit from a more customized knee arthroplasty.

The aim of this narrative review about personalized medicine in arthroplasty patients was to highlight the individual anatomic features that might lead to dissatisfaction, if they are modified outside the range of their limits because of surgical compromise. This can be because of a change in the constitutional alignment in all three planes, a lack of respect for the anthropometric features of each person or secondary symptoms, such as bone overload and subsidence, subtle or gross instabilities and overstuffing of different compartments.

In this narrative review, the authors will discuss indications for unicondylar, patellofemoral, bicompartmental, as well as customized total knee arthroplasty.

## Materials and methods

A Pubmed and Google Scholar search was performed in January 2021 by two authors (JB & MKM) with the following terms: “patient-specific knee” followed by adding “and arthroplasty” and “custom implant” and “total knee replacement” and “partial knee replacement” and “patellofemoral knee replacement” and “bicompartmental knee replacement”. This lead to an initial 1029 papers that were found, reduced to 709 and 57 articles. The search for PKR and PFR lead 1085 studies to and bicompartmental knee replacement to 57 results. When in doubt to include a study a third author (ET) gave his advice and a majority choice was followed. After reading the abstracts and eliminating doubles, 90 papers remained and were considered useful for this narrative review. No risk of bias analysis was performed for the included studies.

## Results

### Unicondylar knee arthroplasty

#### Indications

The main indication for unicondylar knee arthroplasty (UKA) is mono-compartmental Kellgren–Lawrence stage IV osteoarthritis with anteromedial bone-on-bone contact [17]. The strict contraindications for UKA as described by Kozinn and Scott in 1989 [18] were attenuated over time with more evidence-based indications [19, 20]. The anterior cruciate ligament (ACL) needs to be functionally competent. In medial osteoarthritis (OA), an anterior location of the OA indicates that the ACL is intact. In contrast, a posterior location of OA indicates ACL deficiency [21]. Osteophytes within the other compartments can often be ignored if the joint space, and therefore the cartilage height, is completely intact in stress views or on ArthroCT [22]. A decreased joint space height of the other compartment is a contraindication for UKA, since this can lead to disease progression and revision [23]. For medial UKA, the medial collateral and anterior cruciate ligament have to be intact [19], the varus deformity has to be reducible so that on valgus stress radiographs a normal joint space height is visible. A coronal deformity of more than 15°, because of intra-articular wear, should also be considered a contraindication [24]. Medial patellofemoral OA without bone loss is no contraindication for isolated fixed or mobile bearing [25, 26], but it has been proven that lateral facet OA of the patella can lead to lower satisfaction rates and, therefore, might ultimately lead to revision [27]. The reason for this can easily be found in the coronal and axial realignment obtained by the intra-articular correction of the deformity during the resurfacing procedure of UKA. Flexion contracture over 10° has been a contraindication for a long time, but a recent study showed that in a cohort with flexion contractures of up to 20°, improvement of functional scores was higher than after TKA, despite that some flexion contracture might remain after surgery. In this regard, more and better data are necessary for profound decision-making [28]. Obesity is no contraindication for performing UKA [19]. Even in patients with a BMI over 35 no inferior ten-year survival was found and especially this group showed the greatest improvement in Oxford Knee Scores (OKS) [29]. Chondrocalcinosis did not affect survival or functional outcome of UKA and is, therefore, not a contraindication, even if the other compartment is affected without cartilage loss [19, 30]. Age under 60 is associated with higher revision rates, but also with higher satisfaction rates compared to older patients. The main reason is thought to be a higher activity level of these patients. This has to be clearly discussed with the patient [24, 31]. Diffuse pain can occur in unicompartmental osteoarthritis due to diffuse synovitis and is not a contraindication for UKA [24]. Inflammatory disease or septic arthritis is clear and undebated contraindications.

The most important issue for good outcomes in a personalized arthroplasty program is to exclude patients with contraindications for that surgery [32].

### Outcome and survival comparing TKA and UKA

A randomized controlled trial of 528 patients, randomly assigned to TKA or UKA, found no differences in survival, clinical outcomes, and revision rates at 5-year follow-up. However, length of hospital stay and effective costs were significantly lower in the UKA group. Patients answered more often yes to the question if they would undergo surgery again in the UKA group. A recent review of 60 studies, reported significantly better functional outcome scores for UKA, while pain did not differ between both groups. Furthermore, a higher mortality rate was reported for TKA [12, 33].

In contrast, a large registry study of more than 100 000 matched patients comparing TKA and UKA found worse implant survival and a higher overall revision rate of UKA at 8 years [12]. However, registry-based data have the enormous bias of recording all implantations irrespective of the number of cases per surgeon.

A study of a high-volume centre with 2 years of follow-up, randomized patients to a simultaneous two team bilateral TKA or UKA procedure. The authors reported similar outcome regarding function, activity level and satisfaction, but also significant faster rehabilitation, less complications and less early revisions in UKA [34].

Lateral UKA is performed less frequently than medial UKA, but it is a good solution for isolated lateral disease. The reason for the lateral compartment, not to develop OA as often as the medial compartment, might be the biomechanical force distribution of 40–60% with a higher load of the medial compartment [35]. Therefore, high-grade lateral OA is mostly posttraumatic due to fractures or after total meniscectomy. Implant survival has been reported comparable to medial UKA at 5, 10 and 15 years postoperatively [36]. No high-quality studies, i.e. randomized controlled trials, are available to compare medial to lateral UKA. The results of the many retrospective case series are partly controversial. Overall similar revision rates and subjective outcomes to medial UKA can be found [37]. Since the most reported complication leading to revision is dislocation in mobile bearings, fixed bearings should be the first choice for lateral UKA. The reason for higher bearing dislocation rates, in lateral than in medial UKA, is mostly seen in the naturally looser lateral flexion gap that can show inter-individual differences [37]. Mobile bearings showed biomechanical advantages, but these theoretical advantages were not reflected in the outcome of a randomized controlled trial with a 2-year follow-up [38]. The ideal indication for a lateral UKA, is a Kellgren–Lawrence stage IV osteoarthritis, often only seen on a Lyon schuss or Rosenbergs view, because the disease is posterolateral [39]. Restoration of the patient’s individual anatomy is the goal of contemporary arthroplasty. Lateral UKA, as a resurfacing procedure, typically restores the patient’s own morphotype, by the principle of epiphyseal resurfacing and, therefore, undercorrection, often leading to a better functional outcome [40].

The choice between UKA and TKA can often be a dilemma between function and implant survival as stated by Thienpont and Baldini in their editorial [41]. UKA’s are revised threefold more than TKA’s, but if we exclude the most frequent causes of revision such as aseptic loosening, polyethylene dislocation and infection, the other indications are often subjective and ad librum of the opinion of the revising surgeon. Registers show that disease progression, malalignment and unexplainable pain remain frequent causes for revision. If real bone to bone OA would be visualised in the contralateral compartment because of disease progression, two options exist. The most conventional one would be to revise to a TKA, but with the same success another UKA component could be added, respecting the central pivot and its stability [42].

UKA can also be a personalized solution for the elderly and more fragile patient. Since less blood loss [43] and less co-morbidity and mortality have been observed [12]. For those surgeons who believe they should identify the best patient-specific solution for each individual patient, UKA should certainly be one of their technical solution for mono-compartmental OA [44].

The past of UKA has shown that the tibia is the difficulty to tackle with regard to implant fit. Conventional unicondylar systems usually offer only a medially mirrored model for the lateral compartment. The anatomical differences between the medial and lateral tibial plateau are not taken into account. The use of a custom UKA or a morphometric implant [45] can be the solution. Patient-specific instruments can easily help with accurate cuts, avoiding too deep posterior cuts or undercutting of the spine [46]. Patient-matched anatomical implants avoid over- and underhang, as well as subsidence or proximal tibial pain. A study reported significantly worse Oxford knee scores and pain scores 5 years postoperatively with tibial component overhang greater than 3 mm [47]. This study has emphasized the importance of respect for the tibial anatomy and the need to avoid impingement of the medial collateral ligament.

### Patellofemoral arthroplasty

Isolated patellofemoral arthritis is found in 9% of all people over 40 years of age [48]. Women are affected more frequently and suffer from isolated patellofemoral arthritis in 24% of cases when gonarthrosis is present [49]. Proper patient selection is crucial. Hence, a detailed clinical examination, standard radiographs in three planes, a Rosenberg view and full leg standing radiographs are advised. MRI or ArthroCT to assess tibial tuberosity to trochlear groove (TTTG), as well as the quality of the cartilage in the other two compartments can also be recommended [22].

Patellofemoral joint arthroplasty (PFA) is the therapy of choice for isolated symptomatic osteoarthritis of the patellofemoral joint after unsuccessful conservative therapy when bone to bone OA Iwano IV is observed. The best indication is secondary OA due to patellofemoral dysplasia. Posttraumatic OA, after multiple surgeries of the patella or realignment procedures might be more difficult to treat with great patient satisfaction [50].

Contraindications include tibiofemoral instability, high-grade tibiofemoral arthritis (Kellgren-Lawrence 3 or 4), malalignment, infection, rheumatoid arthritis and ankylosis of the knee joint. The limits are an extension deficit > 10°, as well as a reduced flexion arc (< 90°) [51]. Following these strict indications, TKA can usually be avoided in isolated patellofemoral osteoarthritis, since young patients are mostly affected.

Two different systems are available for the trochlea: inlay or onlay prostheses. With inlay prostheses, only the defective cartilage of the trochlea is removed and milled out. This results in minimal bone loss and retention of the thickness of the trochlea, as the prosthesis is implanted at the level of the healthy cartilage. Reconstruction of the desired trochlea shape is achieved using implants with different degrees of curvature. Inlay prostheses are intended to prevent overstuffing of a normal trochlear anatomy [52]. However, in the case of rotational errors or axial deformities, additional procedures are often necessary to obtain accurate patellar tracking. Onlay prostheses, based on the concepts of TKA, require complete resection of the trochlea and are not dependent on the previous shape of the trochlea, so they can be used to treat and correct higher grades of dysplasia. Rotational defects, genu valgum or genu varum (within 3°) are not a contraindication and the alignment of the prosthesis can correct the patellofemoral tracking. There is increased bone loss and risk of overstuffing in comparison to inlay prostheses [53].

Inlay design showed a higher failure rate in the Australian registry compared with the onlay design, because of patellar maltracking, pain and clunck phenomenon [54]. Two studies from 2019, concerning the same inlay implant, obtained controversial results. While one showed promising results with a survival rate of 91% at 2 years and 83% at 5 years [55], the other found a high failure rate of these inlay prostheses [56]. Many patients had to be revised after 28 months and converted into an onlay prosthesis because of persistent pain and clunck phenomenon. Failure occurred mainly in patients with a high Insall–Salvati index, patella alta and craniolateral osteoarthritis. The choice for an inlay prosthesis is therefore described as a relative contraindication in these cases [56].

In comparison to TKA, PFA has been described superior in terms of knee function, postoperative activity scores and recovery time [57, 58]. As mentioned above, the higher failure rates compared to TKA in registry data [13] needs to be levelled out with the more promising results of centres with a higher number of implantations. Ackroyd et al. examined 85 patients with 109 patellofemoral prostheses in a retrospective study and demonstrated a 95.8% survival rate after 5 years. Twenty-eight percent of patients had radiographic progression of osteoarthritis [59]. Another study showed that 75% of implanted first-generation prostheses were still functional after a mean follow-up of 10 years [60].

The main reason for revision surgery is obesity associated with coronal malalignment because this can lead to rapid progression of osteoarthritis in the tibiofemoral compartments. In these patients, primary implantation of a TKA should be considered what allows to correct their alignment issues [61]. The true indication for PFA is a patient with tibiofemoral hypertrophy of the cartilage in the presence of PFOA secondary to dysplasia [22].

A recent study showed that PFA is economically more favorable than TKA after 1 year of follow-up. The burden on health care systems can also be an important argument nowadays, within the therapeutic options. Further, good long-term survival of PFA has been reported with a survival ranging from 70 to 86% in a follow-up period ranging from 10 to 20 years [62, 63].

PFA seems to be preferable to TKA for well-selected patients with isolated patellofemoral arthritis. Third generation onlay designs allow the surgeon to change the dysplastic anatomy of the patient with recessing of the anterior aspect of the femur, a more open proximal trochlear angle and external rotation of the femoral component helping with patellar tracking and patellofemoral stability [50, 64].

Up to date, patient-specific PFA is not available. However, the low implantation rates of PFA and the low volume per surgeon, which is reflected in the controversial results of register studies and studies from high-volume centers, show that a patient-specific approach with patient-specific instruments for a higher surgical accuracy and patient-specific implants for an improved component fit, has great potential.

### Bicompartmental knee arthroplasty

Thirty percent of patients treated with TKA have bicompartmental osteoarthritis [65]. The indication is often OA of the tibiofemoral compartment Kellgren–Lawrence stage IV combined with OA of the lateral patellar facet Iwano stage IV [66].

Today, bicompartmental solutions consist of a modular combination of UKA and PFA with two separate implants, because monobloc systems showed high complication [67] and failure rates [68]. This modular option requires a certain expertise of the surgeon when aligning the implants. Engh et al. compared 50 BKA with TKA in terms of restoring knee function. Differences in Knee Society Score and the Oxford Score were not significant. Functionally, there were initially advantages in favour of BKA, but this evened out after 2 years [69]. After a mean follow-up of 31 months, there was a postoperative improvement in Knee Society and Function Scores, KOOS, SF-12 and WOMAC, as well as in radiographic assessment and implant survival in another study. Of the 29 implanted bicompartmental prostheses, conversion to TKA was required in only one case after 3 years [70]. After a follow-up of 3.8 ± 1.7 years, there were better results for daily activities as well as better function in patients with BKA compared to TKA [71]. A literature review showed that BKA with modular components achieved good to excellent results after a follow-up of ± 10 year. Especially, function and biomechanics are better compared to TKA [72]. However, BKA shows higher failure and revision rates compared to TKA [13]. Besides the low implantation number per surgeon and the complexity of the procedure, implant fit might also be a reason for this. Individual planning of the prostheses could provide an optimal fit and simplify the procedure. Implantation of medial or lateral UKA in combination with a PFA is possible. In a multicenter study, 78 patients received an individual custom BKA. Improvements in knee mobility, as well as in knee scores (KSS and KOOS) were observed. Ninety-seven percent of implants survived after 2.6 years [73]. Ogura et al. could confirm these results, showing a survival rate of 98% after 2 years and 92% after 5 years. The WOMAC, VAS and SF-36 scores showed significant improvement and the patients were satisfied with the results [74]. The implantation of a custom BKA is promising. However, the data are manageable so far and longer follow-ups are necessary.

### Patient-specific TKA

When patients need a TKA because of fixed deformities, important angular deviations [75] or the absence of ACL or PCL [21], the authors believe that the individual anatomy of the patient will be modified in many more ways than with the previous resurfacing type procedures. This has led to many different observations about potential causes for patient dissatisfaction observed in about 20–30% of the TKA population.

Shape mismatch has been considered a first cause of dissatisfaction and this has pushed the industry to develop different options for femoral and tibial components with gender specific implants (standard/narrow components) and addition of numerous (intermediate) sizes. Despite of this step forward with these morphometric implants, resulting in important inventory, imperfect component fit is still found when off-the-shelf implants are used. Several studies describe an association between persistent pain and worse functional outcome with component overhang [47, 76–79]. Femoral component overhang of more than 3 mm can be observed with a relatively high frequency, in particular in female patients. In these patients, the odds ratio for significant postoperative pain was doubled 2 years after surgery [78]. A CT study showed that up to 25% of the study cohort, especially patients with high anteroposterior and mediolateral dimensions, would have a mismatch between the bony anatomy and the femoral component of 3 mm if they would undergo surgery with a modern femoral component [80]. Intraoperatively, there are not many options as to choose a smaller implant size, if the component exceeds the bone if a standard OTS system is used. Downsizing may have a negative impact on the clinical outcome. Mediolaterally, cancellous bone that is not covered by the component, represents a risk for increased postoperative bleeding [81]. Anteroposteriorly, the posterior offset of the femur is decreased with an anterior referencing system, causing the tibial component to impinge with the femur earlier during roll back, which may result in decreased flexion capability [82]. With a posterior referencing system, this will lead to anterolateral notching depending of the external rotation [83].

A study on 24,042 CT data showed that 80% of the study cohort had a difference of more than 2 mm between the two posterior offsets [80]. However, most off-the-shelf systems do not take the difference between medial and lateral posterior condyle into account due to their mostly symmetrical design, which may lead to excessive medial bone resection, external rotation of the femoral component to achieve a balanced symmetric flexion gap and soft tissue releases, if mechanical alignment is used. Similar considerations must be made with the distal condylar offset to achieve a balanced extension gap [80]. Implants addressing this almost standard asymmetry were recently developed on femoral and corresponding inlay side. However, despite this modern and reasonable approach they again just offer a fixed amount neglecting the huge variability. Keeping in mind the findings of the huge database of > 24.000 CT scans finding asymmetry as high as 8 mm [80].

Similarly, manufacturers introduced asymmetric designs to better replicate tibial anatomy. These designs have a fixed degree of asymmetry and a fixed ratio of mediolateral and anteroposterior widths, often increasing with implant size. However, another important CT study reported a high variability of tibial asymmetry that cannot be adequately addressed by off-the-shelf asymmetric designs [84]. Besides, some designs having symmetric anteroposterior dimensions. Using a symmetric component on an asymmetric tibia plateau results in either posterolateral overhang or posteromedial undercoverage, while the use of an asymmetric component on a symmetric tibial plateau leads to posterolateral undercoverage or posteromedial overhang. Furthermore, these compromises also affect and often negatively impact another important issue of tibial implant fit, namely implant rotation. Implant manufacturers continue to introduce more sizing options and alternative designs to address the anthropometric and gender differences. Narrower and wider femoral implants may address coverage issues and limitations in aspect ratio (AP/ML). Keep in mind the findings of a huge database of > 15.000 CT scans that only 14% of tibiae exhibited symmetric (< 2 mm offset) lateral and medial plateaus, and 22% had an offset > 5 mm [84].The problem for these new morphometric knee designs lies in the limited amount of patient-specific knee analyses that were performed to determine the shape of their future components. Furthermore, the shape of femur and tibia depends of the resection level, angles of correction and the rotation planned for that individual patient. More external rotation of the femur, with a symmetric implant, leads to either anterolateral notching or the use of a bigger AP size of femur [83]. A patient-specific rotation matches the original anatomy combined with a unique aspect ratio, guaranteeing perfect coverage. Already in 2014, an editorial entitled anthropometric measurements of the knee: time to make it fit [85] called for more implant variability. Last but not least, the slope of the tibia is different between the medial and lateral side with an intra-individual mean of > 2° and differences as high as > 9° [86]. So any tibial plateau design, with symmetrical polyethylene conformities, can only be an approximation of the individual anatomy.

With a customized total knee design, the component matches the patient’s anatomy to ensure precise fit. In addition, the medial, lateral, and trochlear J-curves are anatomically reconstructed and the patient’s native distal and posterior femoral offset is preserved. These design principles restore the relative position of ligament origin and insertion and therefore reduce the need for ligament balancing, but especially subtle instabilities in the three planes by modifications of the different ligament isometries.

Several studies reported better component fit of customized implants, including superior cortical bone coverage [87], less need for femoral and tibial bone resections [88] and optimal tibial rotation while maintaining proper bone coverage [89], compared to off-the-shelf implants.

Concerning knee kinematics, it has been reported that customized implants achieve kinematics that are more close to those of the native knee joint compared to off-the-shelf implants [90–92]. A study investigating contact stresses on the polyethyene using finite element models reported that customized implants had decreased contact stresses under gait cycle conditions [90].

Regarding clinical outcome, several studies reported on better function compared to off-the-shelf implants [74, 93, 94], resulting in higher patient satisfaction [74, 93]. Manufacturers of off-the-shelf implants added more implant sizes including narrow options to optimize the implant fit. It has been shown that this reduced the prevalence of femoral component overhang especially in female and Asian patients [95]. However, the costs of offering multiple implant sizes coming along with re-processing costs of multiple trays of instruments and implants [96] should be balanced against the initial higher costs of customized implants. Besides the re-processing costs, multiple trays may reduce the efficiency leading to an increased OR time and also longer turnover times between cases [96]. By reducing the amount of implants and instruments needed, customized implants can reduce turn over time. By reducing surgical steps, customized implants can reduce OR time, which is beneficial for the patient [96]. In addition it has been shown, that the use of customized implants can achieve savings in comparison with off-the-shelf procedures, driven by lower initial costs and lower postoperative spendings for inpatient services [97].

Even though it might be desirable to offer a customized implant to each patient to implement the approach of personalized medicine, there are patients that may benefit more from a customized implant than others. These patients have high anteroposterior or wide mediolateral dimensions, with differences in posterior and distal femoral offset and different degrees of tibial asymmetry.

Despite of the yearlong focus on concepts like coverage and avoidance of overhang, other anatomic variances, such as overstuffing and three plane joint line changes can also affect function and patient satisfaction [98, 99]. The optimal coronal alignment of TKA is still under debate and is reflected in the various alignment philosophies discussed today, such as mechanical alignment, anatomical alignment and kinematic alignment. In the past, surgeons aimed for a neutral mechanical alignment, because it was obtained most consistently with conventional instruments and it had been shown, that mechanical peak forces increase with incremental varus positioning of the tibial base plate [100]. However, opponents argue, that for a group of patients (i.e. patients with constitutional varus), neutral alignment is abnormal [101]. Alterations of the level and angle of the joint line can result in abnormal contact kinematics. Correction of coronal deformities requires soft tissue releases and the desire to avoid these has led to a growing interest in kinematic alignment in the recent years. Kinematic alignment aims to restore the individual pre-arthritic alignment [102]. However, complex and severe alignment deformities exist with intra- and extra-articular origins [21], and pre-arthritic alignment may differ from arthritic alignment. A recent review reported that current TKA alignment philosophies do not sufficiently address the high coronal variability in osteoarthritic knees [103]. This is further emphasized by a study who identified 162 different functional knee phenotypes in a CT study including 2764 osteoarthritic knees. They concluded that a more personalized alignment strategy in TKA is needed and that the challenge lies in identifying the optimal alignment strategy for the individual patient [104].

To allow a fully patient-specific approach, the individual knee anatomy including shape, alignment, kinematics and kinetics needs to be respected for all its diversity.

## Conclusion

Dissatisfied patients and the continuous surgical compromises encountered during conventional TKA highlight the need for more personification in orthopaedics and more specifically in joint arthroplasty. A one-fits-all approach with conventional TKA is outdated. The disease should be treated with resurfacing procedures only where the disease actually is present. The combination of correct indication, customization of implants, preoperative 3D planning and patient-specific instruments has the great potential of improving patient satisfaction. Initially higher costs should be leveled with savings over time due to potential lower adverse event rates.
